# Obstetric shift-to-shift handover in Kerala, India: A cross-sectional mixed method study

**DOI:** 10.1371/journal.pone.0268239

**Published:** 2022-05-12

**Authors:** Lucy Pilcher, Merina Kurian, Christine MacArthur, Sanjeev Singh, Semira Manaseki-Holland

**Affiliations:** 1 Institute of Applied Health Research, College of Medical and Dental Sciences, University of Birmingham, Birmingham, United Kingdom; 2 Amrita Institute of Medical Sciences, Kochi, Kerala, India; Flinders University, AUSTRALIA

## Abstract

**Introduction:**

Beyond the provision of services, quality of care and patient safety measures such as optimal clinical handover at shift changes determine maternity outcomes. We aimed to establish the proportion of women handed over and the content of clinical handovers and communication between shifts within 3 diverse obstetrics units in Kerala, India, and to describe the handover environment.

**Methods:**

A cross sectional study was conducted for six weeks during February and March 2015at three hospitals in Kerala, India, during nurses obstetric handover in one tertiary private, one tertiary government and one secondary government hospital. Nursing handovers in obstetric post-operative, in-patient and labour wards were sampled. An SBAR-based (situation, background, assessment and recommendation) data schedule was completed whilst observing handover at nursing shift changes. Since obstetricians had no scheduled handover, qualitative interviews were conducted with obstetricians in two hospitals to establish how they acquire information when beginning a shift.

**Results:**

Data was obtained on 258 patients handed over, within 67 shift changes. The median percentage of women handed over was 100% in two of the hospitals and 27.6% in the other. The median number of information items included out of a possible 25 was 11, 5 and 4,and did not change significantly for women with high-risk status. Important items regarding assessment and recommendation for care were often missed, including high-risk status. The median number of environment items achieved was good at 7 out of 10 in all hospitals. Obstetricians sought information in various ways when required. All supported the development of structured tools, face-to-face and team handovers.

**Conclusions:**

Maternity unit handovers for doctors and nurses were inadequate. Ensuring handover of all women and including critical information, between shifts as well as between doctors, needs to be improved to increase patient safety.

## Introduction

Sustainable Development Goal (SDG) three targets the decrease of the global maternal mortality ratio (MMR) to less than 70 per 100,000 live births by 2030 [1]. In 2015, after the global improvement in MMR observed due to efforts of Millennium Development Goals, India still had a MMR of 103 per 100,000 live births between 2017–2019 [2]. To reduce complications and MMR rates, women are advised to deliver in health care facilities in low middle-income countries (LMICs). Often it is the high-risk women who are specifically attending to deliver in hospital or to be admitted for observation. However, if patient safety and continuity of care between staff is not ensured maternity care becomes suboptimal which can contribute to maternal and neonatal morbidity and mortality. High quality communication and handovers are essential for patient safety in order to reduce errors in care and ensure continuity of care [3]. Research conducted by members of our team in non-communicable diseases in India in-patients, out-patients and community settings indicated that there is a great need to improve systems at all levels [4–6]. India is a vast sub-continent and the health system in each state varies slightly based on the state’s department of health facilities and the policies which govern the management of hospitals. Variation is also found between the type of institution, for example whether they are private or state-led, secondary or tertiary hospitals and which hospital speciality is being considered. Therefore, a generic handover system in India cannot be summarised here. There are no references to site as such. To further research this theme we have explored obstetric shift change handover as it has the potential to greatly impact patient safety, in addition to maternal and neonatal outcomes. Furthermore, adequate clinical handover has been noted as an essential requirement of optimal maternity care by WHO in their standards document [7].

Clinical handover is a crucial aspect of clinical communication, described as “the transfer of professional responsibility and accountability for some or all aspects of the care of a patient, or group of patients, to another person or professional group on a temporary or permanent basis” [8].This can occur at shift changes in hospitals. The consequences of poor handover include medication errors, repeated investigations and preventable readmissions [8–10].There is currently no gold standard for maternity care handover. The Royal College of Obstetricians and Gynaecologists of UK (RCOG) and the American Congress of Obstetricians and Gynaecologists (ACOG) have acknowledged handover between shifts is a weakness in obstetric care, even in HICs resource-rich settings and have produced a handover guideline [11, 12]. The UK RCOG guidelines recommend the use of “Situation, Background, Assessment and Recommendations (SBAR)” framework for communication of women’s clinical information at shift changes [3, 13].

Within one Indian state and in three different types of hospital we aimed to investigate the proportion of patients handed over between staff during shift changes in obstetric departments, to describe the content of clinical handover communications using the SBAR tool, the environment of handover, and whether this is conducive to a high-quality handovers.

## Methods

### Study design and setting

A cross sectional study was conducted for six weeks during February and March 2015.

The research was conducted in one city in Kerala, India, in three hospitals: a tertiary private teaching hospital (hospital 1), a smaller secondary government general hospital (hospital 2) and a larger tertiary government general hospital (hospital 3).

Hospital 1 and 3 had up to 70 obstetric and post-operative beds, with up to 11 obstetricians. Hospital 2 provided free care at the point of access, had 40 obstetric inpatient beds with six post-operative beds and three obstetricians.

We selected the state of Kerala as the best-case health scenario amongst the Indian states. In India, Kerala had the lowest MMR of 30 per 100,000 between 2017–2019 [2].Kerala had the highest health index of 82.2 in India between 2019–2020 [14].

### Quantitative analysis

All nurses who were part of clinical handover at shift changes gave signed consent to be observed for the study period.

The data schedule (S1 Fig) was designed and piloted. Previous tools were either not appropriate for the setting or for measuring the study objectives and there were no handover guidelines in India. Thus, this data schedule was adapted from the guidelines produced by ACOG and RCOG, providing some consistency for comparison with other studies. Part one recorded details regarding the handover environment; this was completed for each handover session when the shifts changed. Part two was completed for the woman and recorded what information was handed over for each woman. Women were classified as “stable” or not and/or having undergone a “normal labour” or not to enable comparisons of high-risk handovers. A “normal” labour was defined as a vaginal delivery without any complications or instrumental delivery.

The schedules were completed while the handover sessions were observed wherever handovers took place. The handovers were conducted in Malayalam, the state language therefore two translators were used to complete the schedules. They were local translators sourced and employed for the project by Hospital 1. Neither had a medical background; one was a student who had recently completed a psychology degree and the other worked as a translator. They received training in the completion of the data schedule by author LP. The location of handover was varied; it was held in labour rooms, post-operative wards, obstetrics and gynaecology wards and at nurse stations (see description of handovers in results). Shifts changed at 8am, 2pm and 8pm for all hospitals. Observations were conducted twice a day including weekends or public holidays. Shifts were observed according to the availability of the translators, and an even spread of observations times was ensured in hospitals 1 and 2. There was a lower number of handovers observed for hospital 3 due to a delay in obtaining the required permission. Where multiple handovers were due to happen simultaneously, the handover which began first was observed. Field notes were taken by the researcher to enrich the quantitative data in order to enable analysis of facilitators and barriers.

All handovers conducted in the obstetric departments of the study hospitals, by both nurses and obstetricians, regarding obstetric inpatients were eligible for inclusion. In Kerala, there were no midwives as all nurses had midwifery training. Doctors did not have a formal handover in any of the hospitals.

The number of information items (content of handover) included in the observed handovers was analysed by SBAR category: situation (8 items), background (10 items), assessment (3 items) and recommendation (4 items).

Data were analysed using SPSS software. Non-parametric methods were used for comparisons as variables were non-normally distributed; descriptive statistics have been presented as medians and inter-quartile ranges. Statistical significance was defined at p<0.05. A sample size of 97 patient handovers per hospital was calculated using a 95% confidence level and 10% margin of error. In order to estimate the rate of occurrence of items on the data schedule, it was assumed that 50% of handovers would contain all items on the data schedule to produce a conservative sample size. For the multivariate analysis outlined in Table 3, the variables included are shift times, hospital, location within hospital/ward setting, working day and duration.

### Qualitative interviews and analysis

The quantitative and qualitative studies were conducted in parallel. The quantitative data schedule could not be applied to shift-to-shift handovers between obstetricians as unlike the nurses they did not have formal meetings or an alternative system for when shifts changed. Therefore, six individual semi-structured interviews (approximately 20 minutes each) were conducted to understand how the obstetricians acquired information when seeing a woman during a shift, their impressions of the effectiveness of information exchange in these circumstances and possible barriers to improving the system [15, 16]. The interviews were conducted by a single UK medical student (LP) who had training on qualitative methodology. These interviews were conducted in English by author LP, with the assistance of a translator if required and were recorded, transcribed and anonymised [17]. Braun and Clarke’s six step process was followed for analysis [18].

Convenience sampling was used to select three consultant obstetricians from each of hospitals 1 and 2.The sample was determined as there were only 3 obstetric consultants in hospital 2, so this number was matched at hospital 1. No permission was granted to undertake qualitative interviews in hospital 3 so information presented regarding handover in hospital 3 was gained via explanation from nursing staff available at the time of nursing handover observation. A piloted topic guide was used (S2 Fig). Other than a simple description of handover, a thematic content analysis was performed for the other two research questions regarding the opinion of obstetricians about the quality of handover and barriers to optimal handover. This method is fully described in S1 File. 2. Co-author SMH independently coded the transcripts, both LP and SMH coded and agreed on codes and discussed interpretations of the findings [19]. In hospital 3 it was explained that the system was similar to hospital 1 and 2 with no structured or regular handover at shift-changes for doctors. A junior doctor managed the wards changing every24-hours and senior doctors left the hospital at 2 or 3pm while being on-call for emergencies.

### Details of ethical approval

Local ethical approval was granted by the Amrita Institute of Medical Sciences Internal Ethics Committee on behalf of all data collection sites. Ethical approval was granted by the University of Birmingham BMedSci Population Sciences and Humanities Internal Ethics Review Committee.

## Results

**Fig 1:**
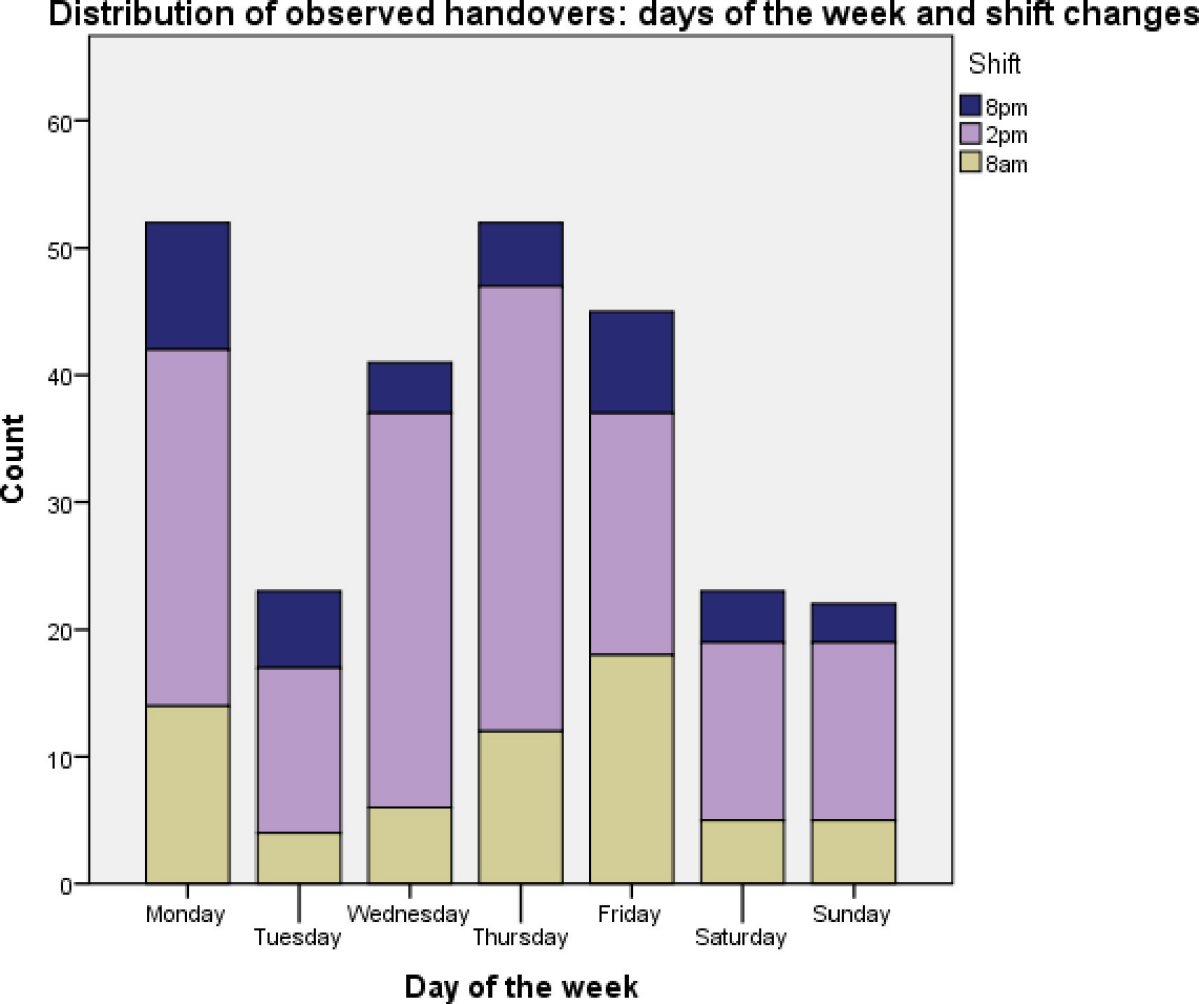
Graph showing spread of observations across days of week and shift changes.

All nurses (37 from hospital 1, 9 from hospital 2 and 4 from hospital 3) agreed to participate in the study. Analysis was performed on the 258 patient handovers from 67 shift handovers in three hospitals (hospital 1 = 121, hospital 2 = 120 and hospital 3 = 17). These were spread across all days of the week and shift handover times from a possible 252 handovers (26.6%) in the three hospitals (in hospitals 1 and 3 mostly between individual pairs of nurses as described below) (Fig 1).

### Description of handovers

In hospitals 1 and 3 nurses handed over the named women they had been responsible for to the named nurse(s) who would take over their care. Therefore multiple handovers occurred simultaneously between pairs of nurses (as one-on-one nurse interactions) around the ward, near to the patients the nurses were responsible for during the shift. In contrast, only one handover took place in hospital 2 as the nurse(s) on shift would be responsible for all women in the department. This occurred at the entrance to the labour ward where the maternity record book was kept.

In hospitals 2 and 3 nurses arrived 30 minutes before their shift started to receive hand over, whereas in hospital 1 the nurses stayed once their shift had ended to perform handover. Nearly all nurses in hospitals 1 and 3 receiving the handover would handwrite brief personal notes about the women handed over to keep on their person throughout the shift. The personal notes were mainly written on small/scrap pieces of paper or folded sheets of paper, with no official proforma.

Hospitals 1 and 3 had patient notes available for handover, whereas hospital 2 used the patient census. The census comprised of a list of all inpatients along with their age, due date, date of caesarean section if planned, plus a sentence or two of additional information about the woman recorded in an A4 notebook with a hard cover, called the Maternity Report Book. Although hospital 1 had an IT system with all clinical patient information, this system was not used for handover.

### Numbers of women handed over

Considering all settings, the median number of women handed over was 5 (IQR = 4) from a median of 12 total women per ward (IQR = 17). The range was 0–100% since some handovers did not take place in one hospital (Table 1). The researcher was told by the nurses that they prioritized the handover of the women they were concerned about.

**Table 1:** Background characteristics of observed handover sessions and individual patient handovers.

	All settings N = 258	Hospital 1 N = 121	Hospital 2 N = 120	Hospital 3 N = 17
**Number of shift changes observed**	67	37	28	2*
**Number of patients nurse responsible for** Median (IQR)	12 (17)	4 (4)	23 (6)	12 (7)
**Number of patients handed over per shift** Median (IQR)	5 (4)	4 (3)	6 (4)	12 (7)
**Percentage of admitted patients handed over per shift** Median (IQR)	75 (72)	100 (0)	27.6 (22)	100 (0)
**Stability of patients** Frequency	76 (29.5)	57 (47.1)	18 (15.0)	1 (5.9)
(%) classified as “stable”				
classified as “not stable”	4 (1.6)	4 (3.3)	0 (0.0)	0 (0.0)
unclassified as “stable” vs. not	178 (69.0)	60 (49.6)	102 (85.0)	16 (94.1)
**Normal vaginal or other deliveries** Frequency (%)	20 (7.8)	10 (8.3)	8 (6.7)	2 (11.8)
classified as “normal labour”				
classified as “non-normal labour”	28 (10.9)	25 (20.7)	3 (2.5)	0 (0)
Unclassified as “normal” or not	81.4%	71.1%	90.8%	88.2%
**Patient handovers per location within hospital** Frequency (%)				
Labour room	56 (21.7)	51 (42.1)	0 (0)	5 (29.4)
Post-operative wards	21 (8.1)	9 (7.4)	0 (0)	12 (70.6)
Obstetrics and gynaecology wards	61 (23.6)	61 (50.4)	0 (0)	0 (0)
All areas handed over together inclusive	120 (46.5)	0 (0.00)	120 (100)	0 (0)
**Patient handovers per shift change** Frequency (%)				
8am	64 (24.8)	38 (31.4)	26 (21.7)	0 (0)
2pm	154 (59.7)	50 (41.3)	87 (72.5)	17 (100)
8pm	40 (15.5)	33 (27.3)	7 (5.8)	0 (0)
**Patient handovers on normal working days (excluding public holidays and Sundays)** Frequency (%)	234 (90.7)	111 (91.7)	106 (88.3)	17 (100)
**Nurses involved in handover** Median (IQR)	2 (1)	2 (2)	2 (1)	2 (2)
**Student nurses involved in handover** Median (IQR)	0 (0)	0 (1)	0 (0)	0 (0)
**Duration of handover in minutes** Median (IQR)	10 (10)	16 (11)	5 (3)	7 (3)

Demographics of all observed shift change handover sessions and individual patient handovers in all hospital settings. Data are frequencies (%) unless otherwise stated.

In the linear regression model two variables were independently and significantly associated with the proportion of the inpatient women who were handed over per shift change (see Table 3): hospital setting and the type of ward (Table 3). Not surprisingly, given the difference between the 3 hospital settings, ‘setting’ accounted for 86% of the variance (adjusted R^2^ = 0.86).

### Handover environment

Considering all patient handovers, the median environment score was 7 out of 10 (IQR = 0, range 5–9) (Table 2). The environment was very similar and good across all settings; some criteria were achieved by all observed handovers. The percentage of observed handovers that complied with the important item “free from distractions” was the lowest in all hospitals (14%).

**Table 2:** Frequency of inclusion of each information item on data schedule (part two: 25 items) and frequency of inclusion of each environment item on data schedule (part one: 10 items).

Item on data schedule	All settings, N = 258	Hospital 1 N = 121	Hospital 2 N = 120	Hospital 3 N = 17
**Total number of items included** median (IQR)	6 (7)	11 (5)	4 (2)	5 (3)
**Situation**				
Patient name	257 (100)	121 (100)	119 (99)	17 (100)
Patient age	163 (63)	120 (99)	39 (33)	4 (24)
Patient location	144 (56)	113 (93)	27 (23)	4 (24)
Brief history	160 (62)	104 (86)	49 (41)	7 (41)
Advanced directive or resuscitation status	2 (1)	2 (2)	0 (0)	0 (0)
Key patient values and preferences	26 (10)	24 (20)	2 (2)	0 (0)
Brief expression of concerns	40 (16)	32 (26)	7 (6)	1 (6)
Detailed expression of concerns	19 (7)	19 (16)	0 (0)	0 (0)
Special patient needs	1 (0)	0 (0)	1 (1)	0 (0)
**Background**				
Date of admission	154 (60)	98 (81)	53 (44)	3 (18)
Current medications	200 (78)	111 (92)	76 (63)	13 (77)
Allergies	7 (3)	5 (4)	2 (2)	0 (0)
Diagnosis/active problem list	76 (29)	66 (55)	10 (8)	0 (0)
Results of physical examinations	86 (33)	66 (55)	14 (12)	6 (35)
Progress during admission	92 (36)	67 (55)	18 (15)	7 (41)
Laboratory results	155 (60)	94 (78)	51 (43)	10 (59)
Pending test results	44 (17)	32 (26)	9 (8)	3 (18)
Other information from patient charts	60 (23)	56 (46)	0 (0)	4 (24)
**Assessment**				
Vital signs	101 (39)	75 (62)	20 (17)	6 (35)
Clinical impression	29 (11)	27 (22)	1 (1)	1 (6)
Critical assessment of situation	38 (15)	29 (24)	6 (5)	3 (18)
**Recommendations**				
Management plan	69 (27)	61 (50)	6 (5)	2 (12)
Anticipated therapy	21 (8)	18 (15)	1 (1)	2 (12)
Suggestions/specifics about requests	10 (4)	10 (8)	0 (0)	0 (0)
Suggestions/specifics about timeframe	9 (3)	9 (7)	0 (0)	0 (0)
**Frequency of inclusion of each environment item on data schedule (part one: 10 items)**	86 (33)	66 (55)	14 (12)	6 (35)
**Item on Data Schedule**				
Handover not delayed (except in emergencies)	258 (100)	121 (100)	120 (100)	17 (100)
Junior doctor or nurse participation	258 (100)	121 (100)	120 (100)	17 (100)
Only standardised medical language was used	258 (100)	121 (100)	120 (100)	17 (100)
Hard copy of information alongside verbal handover	258 (100)	121 (100)	120 (100)	17 (100)
Opportunity for receiving team to ask questions	254 (98.5)	118 (98)	119 (99)	17 (100)
Information repeated back to ensure accuracy	242 (93.8)	112 (93)	113 (94)	17 (100)
Primary person or team responsible for each patient identified	214 (83)	121 (100)	88 (73)	5 (29)
Handover was not overheard by those not involved	53 (20)	20 (17)	33 (28)	0 (0)
Handover was free from distractions	37 (14)	3 (3)	22 (18)	12 (71)

Frequency of inclusion of each item during each patient handover and frequency of inclusion of each item during each patient handover, in descending order of frequency for all handovers, in all hospital settings. Data are frequencies (%) unless otherwise stated.

The logistic regression model for a handover being free from distractions accounted for 53% of the variance (Nagelkerke R^2^ = 0.53) and four variables were significantly associated: time of shift, duration of shift handovers, type of ward, and hospital (Table 3). The least distraction occurred at 8am. Another logistic regression analysis of handovers that were not overheard found that the same four variables were significantly associated (Table 3). The model accounted for 26% of the variance (Nagelkerke R^2^ = 0.26).

**Table 3:** Multivariate analysis of variables associated with the percentage of patients who were handed over, variables associated with the total number of information items included in handover, variables associated with handovers which were free from distractions, variables associated with handovers which were not overheard.

		Percentage of patients who were handed over*		The total number of information items included in handover**		The variables associated with handovers which were free from distractions***		Variables associated with handovers which were not overheard****	
Variables*	**Comparison group**	**Coefficient (B)**	**P-value**	**Coefficient (B)**	**P-value**	**Odds ratio (95% CI)**	**P-value**	**Odds ratio (95% CI)**	**P-value**
Shift times	8am vs. 2pm shift change	-1.729	0.408	-0.814	0.029	3.870 (1.410–10.621)	0.009	3.148 (1.428–6.941)	0.004
	8pm vs. 2pm shift change	-0.467	0.861	3.759	<0.001	0.00 (0.00–0.00)	0.997	11.287 (3.905–32.626)	<0.001
Hospital	Hospital 2 vs. hospital 1	-71.401	<0.001	-5.576	<0.001	0.402 (0.690–2.335)	0.310	7.173 (1.518–33.888)	0.013
	Hospital 3 vs. hospital 1	-0.687	0.881	-5.373	<0.001	25.635 (4.212–156.025)	<0.001	0.00 (0.00–0.00)	0.998
Location within hospital/Ward setting†	Post-operative setting vs. labour room	0.332	0.938	1.301	0.840			0.00 (0.00–0.00)	0.998
	Ward setting vs. labour room	-6.182	0.018	-0.587	0.202			6.048 (1.753–20.874)	0.004
Working day	Working day vs. Sundays and public holidays)	2.578	0.387	0.001	0.998	1.397 (0.368–5.297)	0.62	0.722 (0.261–1.998)	0.530
Duration	Duration of handover as a continuous variable (minutes)	-0.098	0.581	0.073	0.020	0.620 (0.488–0.788)	<0.001	0.961 (0.894–1.033)	0.277

*Multivariate analysis performed using the variables listed in column one; shift times, hospital, locating within hospital/ward setting, working day, duration.

** Multivariate analysis performed using the variables listed in column one; shift times, hospital, locating within hospital/ward setting, working day, duration.

*** Multivariate analysis performed using the variables listed in column one; shift times, hospital, working day, duration. Location within the hospital was not included.

**** Multivariate analysis performed using the variables listed in column one; shift times, hospital, locating within hospital/ward setting, working day, duration.

† Hospital 2 nurses handed over all shifts together in one meeting- therefore this analysis only involved hospital 1 and 3.

### Content of handovers

The median number of information items included in each patient handover was 6 (IQR = 7;range 2–21) from a possible 25. The median number of items included was higher for hospital 1 vs hospitals 2 and 3 (Table 3). Items on the data schedule that concerned patient data (e.g. current medications) were included in patient handover more often. However, some items such as the presence or absence of allergies was rarely mentioned. The information items which required a judgement to be made about the woman (e.g. clinical impression) or to consider the woman in a holistic way (e.g. special patient needs) were included less often. No observed handover included all items on the data schedule.

The linear regression model accounted for 71% of the variance in the number of information items handed over (adjusted R^2^ = 0.71), with three variables significantly associated: time of shift, duration of handover, length of stay and hospital (Table 3).

### High-risk women

Women’s’ ‘stability status’ was only mentioned for 80 (31%) women. There was no significant difference in the information items handed over between the ‘stable’ and not groups(median number of items was 9 (IQR = 8) vs. 10 (IQR = 6) respectively). Whether a woman had a “normal” labour or not was handed over for 48 (19%) women post-delivery; the increase in the median number of items handed over was not significantly different between these two groups either (median number of items was 8 (IQR = 7) vs. 10 (IQR = 6) respectively.

### SBAR technique

For all hospital settings the categories “situation” and “background” had a higher median number of items included in patient handovers than “assessment” and “recommendation” with a large number of patients having no handing over regarding any items in “assessment” and “recommendation” in all hospitals and across all wards (Table 4). No handovers included all items in any categories.

**Table 4:** Descriptive statistics for each category of SBAR technique.

	All settings, N = 258	Hospital 1 N = 121	Hospital 2 N = 120	Hospital 3 N = 17
**Situation (8 items)**				
Median items handed over (IQR)	3 (2)	4 (2)	2 (1)	2 (2)
All items handed over (%)	0 (0)	0 (0)	0 (0)	0 (0)
No items handed over (%)	0 (0)	0 (0)	0 (0)	0 (0)
**Background (10 items)**				
Median items handed over (IQR)	4 (4)	6 (3)	2 (1)	3 (2)
All items handed over (%)	0 (0)	0 (0)	0 (0)	0 (0)
No items handed over (%)	3 (1)	0 (0)	3 (3)	0 (0)
**Assessment (3 items)**				
Median items handed over (IQR)	0 (0)	0 (1)	0 (0)	0 (0)
All items handed over (%)	6 (2.3)	6 (5)	0 (0)	0 (0)
No items handed over (%)	201 (78)	74 (61)	113 (94)	14 (82)
**Recommendation (4 items)**				
Median items handed over (IQR)	0 (1)	1 (1)	0 (0)	0 (0)
All items handed over (%)	2 (1)	2 (2)	0 (0)	0 (0)
No items handed over (%)	188 (73)	57 (47)	116 (97)	15 (88)

Median, inter-quartile range, minimum and maximum number of information items in each SBAR (situation, background, assessment and recommendation) category included in patient handovers, and frequency and percentage of handovers including all or zero items from each category in all hospital settings.

### Qualitative interviews describing doctor’s handover

Participants 1, 2 and 6 were qualified obstetricians from hospital 2 (secondary government) while participants 3, 4 and 5 were from hospital 1 (tertiary private).

#### Description of doctors’ handover at shift changes

As there were no regular handovers in either hospitals, both hospital obstetricians reported having some form of informal oral handover for women with high-risk or ‘problems’. This could be on the phone or face-to-face as one left and another arrived for a shift. If this did not occur, when the doctor was called to see a patient a range of sources was used to obtain information about the clinical situation of the woman. These sources were case-notes, ad-hoc oral handovers from post-graduates or interns on the wards or via the phone from doctors who had finished their shift, nurses and asking women about their condition and clinical histories.

***Participant 3:* “***They’ll be writing all these things, the information* … *there is a progress, urr,case-sheet‥‥”*
***Participant 1:****“When I take charge of duty*, *I see that case-sheet … and from that I get most of the information and if*, *if something is not clear*, *I ask the patient or the staff regarding that matter‥‥ Information (from doctors) is taken over the telephone”*
***Participant 4:****“Urr*, *sometimes the nurses give over*, *sometimes PGs (post-graduates) who are there*,*… or sometimes I ring up the consultant’s department who has sent the patient to me”*

#### Sources of patient information to obtain handover

Apart from the above ways of sourcing information, when called to see a patient at the time of being on duty the concept of official handover was not familiar to the doctors. There was a feeling that they knew all the patients through reading case-note documents, verbal handovers at the time of patient admission (if the patient was under their care), ward rounds for getting to know other patients, and that they “unofficially” discussed every patient at shift changes when there were issues with a patient. Obstetricians from hospital 1 reported a higher level of handover even though that too was informal and not at set times. This too occurred only for serious cases or active labour and mostly in an ad-hoc way.

***Participant 4:****“(labour ward) we never leave without giving over*. *Because that is dangerous*. *If information is not properly communicated a drug may be given again*, *or some drug may be missed out*. *… If nothing there active in labour room*,*…we’ll just say there is nothing active in labour room*, *or something like that*. *Only if no serious cases*. *… definitely we have to handover this (serious cases) to the person who is joining the duty before we leave*. *And making sure all patients are stable before we leave…we unofficially discuss each and every patient*. *…I think probably because this is a tertiary referral centre*, *we do not take any risk*, *even if it is small‥‥”*

#### Satisfaction with the handover system

On the whole, all doctors were happy with their system and felt it worked, and that no other way was possible in their circumstances. One talked about how a combination of written and oral handover means women’s information is not missed.

***Participant 2:*** *“The same practice exists*, *in government set ups*, *this will do…(laughs) our set up is like this*, *so many patients*, *‥‥if we want change…no need…”*

#### Opinions regarding face-to-face handovers

Despite appearing satisfied with the current handover system, all doctors *preferred* face-to-face handovers since this provided more clarity, better information and allowed for questions. However this was not seen as practical in most cases.

***Participant 2:****“Yeah…face-to-face talk is more helpful*.*”*
***Participant 4:****“ Definitely*. *That (face-to-face handover) makes a lot of difference‥‥ Definitely it is better because there will be no*, *urr*, *mis-match of information*.*”*
***Participant 6:****”Face to face*, *definitely*, *urr*, *I think that’s better‥‥ because I can*, *urr*, *check (ask) whether*, *urr*, *the information’s reliable because the doctor’s there*.*”*

#### Improving the handover system

As to how the system could be improved, participants said that discussion at each shift change and team handovers would be much more preferable. Two commented that in medical colleges it was possible to have team handovers due to the many doctors present:

***Participant 2:*** *“…only in medical college they were used to take that (meet all together at the start of duty to discuss patients)”*
***Participant 1:****“That (whole team handover) is*, *that is*, *good actually… because where between every shift handing over these discussions take place and*, *and they’re handing over each and every patient*, *that will make much difference …”*

#### Benefits of team face to face handover

The benefits of such a handover were understood in terms of efficiency, improving medical knowledge, patient confidence and continuity of care. Examples from two cases from the private tertiary hospital and one case from the government secondary hospital mentioned better patient safety and reduced errors with face-to-face handovers (see previous quotes).

***Participant 2:*** *“that (team handover at every shift) is very good*, …, *ideally we have each and everything to discuss*, *then patient will get a confidence*, *…our knowledge also improves*, *‥but urr*, *time is not available”*
***Participant 1:****“Actually …if we know a patient when I come to the duty*, *I don’t have to recheck all the case-sheets and all*, *…History taking*, *examination*. *Everything will be reduced and I can directly go for the management…can manage much more efficiently”*
***Moderator:*** *How do you think this meeting and transfer of information could affect the outcome for the women?* ***Participant 3:****“Really does*. *Because you know there will be continuous follow-up of the condition of the patient*, *the treatment of the patient*, *everything is there…”*
***Participant 4:*** *“(face-to-face handover) is better… because there will be no*, *mis-match of information‥‥ I told you Rantac 150*, *you heard 100*, *and then you told her 50 and ultimately she gets 25 and actually I told you 150…if it is told to the direct person who is giving the medication or whatever or who is directly managing*, *I think that’s better*.*”*

#### Facilitators for improving doctors’ shift-to-shift handover

The fact that doctors were aware that poor handover led to medical errors and poor outcome for patients is a facilitator to improvement interventions. Furthermore it was recognised that handover via phone calls from doctors on the former shift was not always effective.

***Participant 4:****“(Phone calls is) not always effective*. *… like sometimes they will be busy with some other procedure*. *…if they are good*, *if they are patient*, *they’ll say this is all the drugs we did*, *this is the BP…or suppose sometimes they are out of range (phone signal)*. *Sometimes it is difficult for us to get them*. *But usually what I have seen here in Kerala is that the method of communication is better than in North…”*

#### Barriers to improving doctors’ shift-to-shift handover

However, a potential barrier is that doctors felt that the case-notes were largely comprehensive and adequate for handover.

***Participant 1:*** *“usually*, *most of the information is in the case-sheets*, *so I don’t have to ask more than that …there is much information in here…This is our case-sheet*. *We write important information at the front*, *‥‥this is‥‥this is… … There is now not much to add I think*, *not much to add to this*.*”*

An important theme mentioned by all was that the main barriers to team or face-to-face doctor handover were lack of time and resources and importantly the lack of availability of doctors for handover. In both hospitals, this was caused by either being busy with other duties such as outpatient clinics or emergencies with no time allocated for handover, or delays to arrival of staff, or (in the government hospitals) that doctors would be out of the hospital after early afternoon.

***Participant 4:****”… see the person (doctor) sitting in outpatients attending 80 OPs or 150 Ops*, *they can’t come for everything and tell…”*
***Participant 2:*** *“the only thing is when the person (doctor) who is looking after (the patients) when I come and take over*, *… may be doing some urgent surgery or emergency surgery or thing*, *… but still there is some post-graduate or intern will be there…*
*if some hospitals they meet at the beginning of the duty to discuss the patients*, *umm…you know that type of hospitals means there are so many doctors*, *assistants*, *but here this is a district hospital*, *a small hospital*, *so we will not get time to discuss each and every thing like that…”*
***Participant 6:****“But*, *usually in our set up*, *doctors stay here for say 2–3 o’clock*, *and then*, *then after we get information over the phone only*.*”*

#### Improving handover skills

The final theme was around the need for experience in order to make the handover a good quality; that juniors would only improve their handover skills with experience, but no training was mentioned.

***Participant 3:****“… senior staff … know*, *what is the implication of all these things suppose even an investigation or a physical finding*. *Or*, *whatever it is*, *they know the implication*, *that’s the thing*. *So a senior person giving over is different from a junior person*.*”*

**Fig 2:**
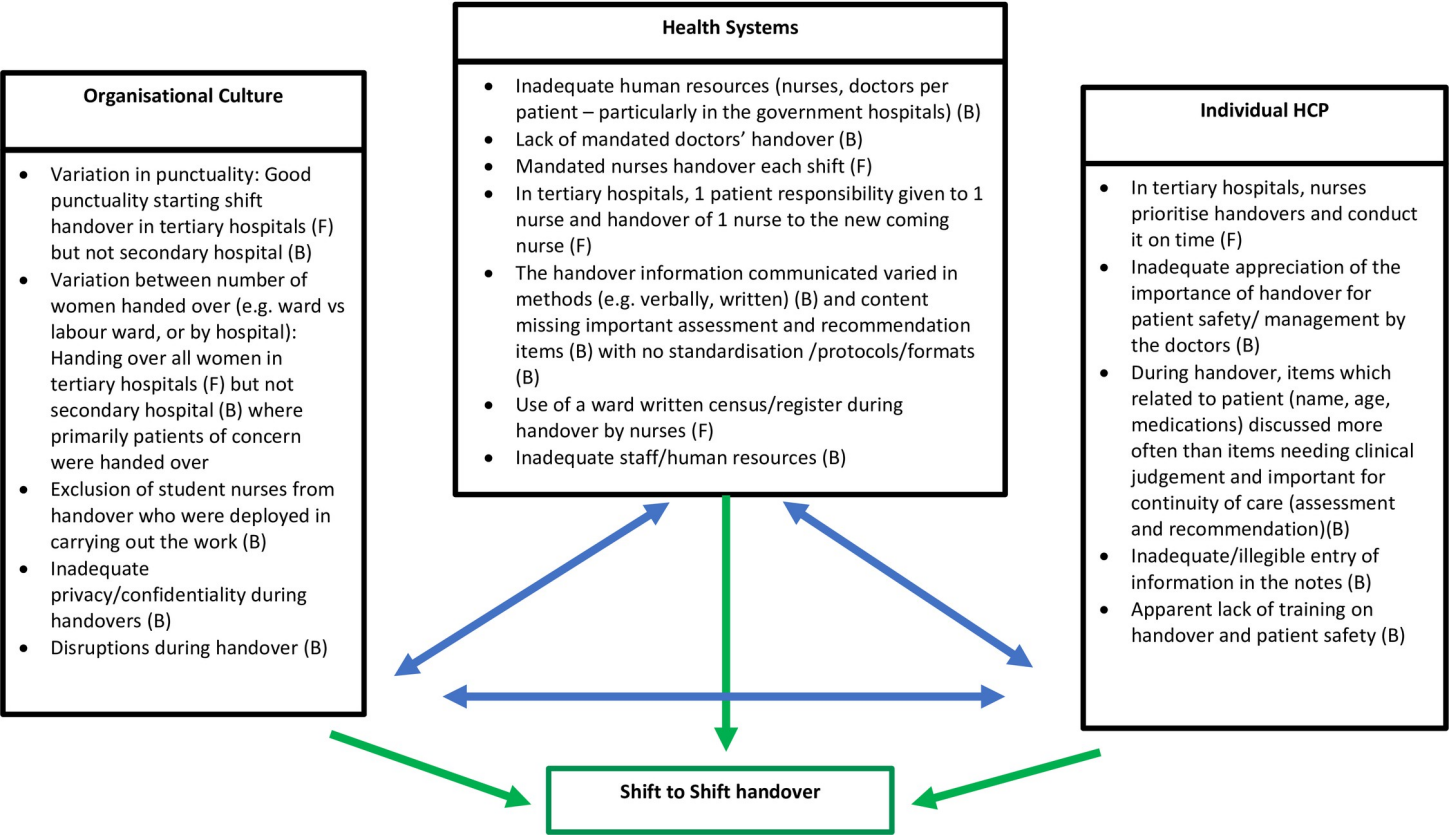
The factors influencing quality of maternity handover. Figure outlining the factors influencing quality of maternity handover in our study. Health systems, organisational culture and individual healthcare professionals (HCP) can affect each other. One factor may have numerous dimensions that can be reflective of multiple influences. Additionally, all three factors influence the shift- to- shift handover. B = Barrier, F = facilitator.

*Triangulation of qualitative and quantitative findings*. Fig 2 represents the triangulation of all findings in the form of a diagram of barriers to optimal shift-to-shift handover in our maternity wards. We have divided each factor influencing quality handover by health systems, organisational culture and individual HCP factors [20, 21]. This can be the basis of a framework for policy and service improvement.

## Discussion

We used an adapted SBAR assessment tool and qualitative methods to investigate shift-to-shift handover in maternity care in three different types of hospitals in Kerala, the state with the best health indicators (S1 File) [14, 22]. We assessed the coverage of in-patients during handover, and the content and environment of handover for nurses who had systematic handovers. We found that in spite of the vastly different types of facilities between hospitals, inadequate and insufficient information was handed over in all settings (median number of information items included of a possible 25 was 11, 5 and 4 respectively) and missing items were critically needed for patient care and continuity. Furthermore, days of the week made little difference to these findings and we observed that numerous staff categories from the multi-disciplinary team involved in patient care were not included in handovers. Importantly, doctors only had informal handovers as and when required through consulting the patient case-notes, talking to nurses, consulting medical students or patients themselves, and telephoning previous shift staff. Doctors’ handover was not prioritised over outpatient clinics or other activities and considered as not possible to achieve in a formal way within the current system; this was the case even in the private hospital where the patient-doctor ratio was (and considered) good. On the other hand in the two tertiary hospitals, a positive finding was that nursing shift-handovers took place on time (Fig 1). Therefore, when considering facilitators and barriers, facilitators for adequate nurse handover were punctuality and nearly universal handover of women in tertiary hospitals. For doctors it was awareness of the general importance of good handover for patient safety and usefulness of face-to-face handover. Barriers included no formal doctors’ handover, non-inclusion of student nurses or interns in nurses’ handovers, inadequate handover of critical information on high-risk status, assessment and management of patient, interrupted handovers and a lack of confidential environment. An apparent lack of formal protocols, tools and training contributed to the above barriers, a finding which was also seen in the general medicine wards in Kerala and in other LMICs [4, 5, 21, 23]. We have categorised these factors in our diagram (Fig 1) to systems, organisational culture and individual healthcare provider factors for easier application to programming [20, 21].

Investigation of clinical handover is important for ensuring high quality patient care, since evidence from high-income countries (HICs) and LMICs indicates that continuity of patient care and thus patient safety are compromised as the result of inadequate shift-to-shift handover [9, 20, 24–27].Other settings advocate the use of SBAR tools for conducting shift-handovers and this proved a useful tool for our assessment [28–32]. On application of an adapted SBAR assessment tool, the number of environment items with which each handover complied was similarly good in all settings. The areas that appeared to be lacking were handovers ‘free from distractions’ and ‘not being overheard’. Most women were handed over between nursing shifts in the secondary care hospital, while tertiary hospitals had more staff and handed over all women by individual designated nurses to new designated nurses arriving on the next shift. The content of the information handed over was the aspect that was inadequate in all settings. Information which related to patient data (e.g. woman’s name, age and current medications) were discussed more often than items which required professional clinical judgment (e.g. SBAR components “Assessment” and “Recommendation”) and those which considered the woman in a more holistic way. The lack of communication of these components was of particular concern due to their relevance to continuity of care and patient safety for women who were in labour, post-operative, or high-risk ward admissions [26–28]. For example, even though in hospital 3 only two shift handovers were observed these were in fully-staffed labour and post-operative wards when optimal handover was expected, therefore scanty handover of ‘Assessment’ and ‘Recommendations’ components was particularly alarming. Significantly, ‘birth type’ or ‘high-risk status’ of women was not always mentioned which would enable closer care of women who required more careful observation.

In other LMICs, data are scarce for all types of shift-to-shift handovers and particularly for maternity care, but conclusions from the three studies we were aware of, were overall consistent with our findings [26, 27]. In the Gambia, similar issues existed with no doctor handover, selective handover of some women and incomplete notes [33]. In contrast to our study in India, high-risk women were more likely to be handed over and with more completed items, while the handover environment was less adequate than in our India study. In Sri Lanka, one study concerning intensive care units noted several issues with handover including no designated time for handover, not all doctors and nurses being present at handover, and a lack of a set structure for the format of handover [27]. Amongst the recommendations, the study suggested that having a printed handover format, a set time and location for handover would be beneficial to improve safety and effectiveness of handovers [27].

In a qualitative patient study performed in Uganda, interviews were undertaken for women as in-patients and following discharge [26].Participants were women admitted for childbirth, due to a complicated pregnancy or delivery [26].The results found that the participants regarded the handovers as being insufficient [26]. Reasons for this included the way in which handover was performed and the inadequate communication to the participants. This contributed to a dissatisfying maternal experience, substandard care and birth trauma [26].The study concluded that there needed to be standardised guidelines for handover, as well as teaching on communication and handover conduct in addition to team working skills for healthcare professionals [26].This resonates with our qualitative findings from the doctors who felt patients would be more satisfied if handovers were more thorough.

As expected, findings in India and LMICs differ from HICs. Nevertheless, maternity handover is still an issue even in HICs. For example, research from a study conducted in the USA classed only 40% of observed obstetric handovers as high-quality [34].Differences in the quality of handover were influenced by several factors such as the time of day of handover[34].Good communication was noted as being important for patient safety [34].

Continuity of care, for example via effective handover and via following handover guidelines, are essential for patient safety [35–40]. A qualitative study conducted in the UK reported that clinicians believed that there should be a set guideline for handover postoperatively to ensure the transfer of key patient information [25]. In our study it was found that there was recognition that high-risk women should not be missed from nursing shift handovers, however, this made little difference to the completeness of information being handed over for these women; an improvement which could be achieved. Qualitative findings demonstrated that some clinicians favoured structured multi-disciplinary handovers to ensure no information was missed-out. Even so, the clinicians felt that there were not enough human resources to enable this to occur despite low cost handover being a health systems quality of care improvement activity [38]. It requires a patient safety culture, handover prioritisation, protocols, training, tools and supervision, and if conducted well, can save time for busy clinicians as well as provide an optimal and safe service.

To ensure a lasting improvement, staff need to be involved in quality of care and patient safety assessments. They should be supported and supervised by senior staff including those in management roles. This would enable a change in organisational culture which prioritises optimal handover. Two HIC studies describe how initial observation of handover by ward staff within specific environments led to the design and implementation of tools which improved handovers [32, 33]. Use of a structured format such as adapted SBAR forms may be useful, although having a structured framework in place still does not guarantee the inclusion of all essential information or the handover of all women [41–43]. Staff training on the guidelines available for safe and effective handover are key, as well as an organisational culture change where patient safety and professional responsibility are reinforced [41]. One hospital patient safety culture survey conducted in a South Indian hospital noted that communication domains still needed strengthening [44, 45]. Involving staff in patient safety assessment and handover improvement may be the key to improved culture. Action towards patient safety has gained special momentum in recent years thanks to WHO, who aim to improve quality of care in LMICs beyond simple provision of care, including promoting the need for effective handover [46]. The WHO has also created maternity care guidelines [7]. Patient Safety was highlighted in 2019 with the dedication of a Patient Safety Day, with handover being a fundamental aspect of patient safety [46].

In addition, evidence suggests that a confidential environment would improve handover [47]. This is possible and easy to achieve in the hospitals participating in our study, if the nurses were to conduct handover in a private location, for example a side room or staff room.

The quality of maternal care provided can vary [48]. Context is vital in health systems improvement interventions such as for handover [49]. To the best of our knowledge, this study is the first to provide such context for interventions to improve obstetric handover in India with possible relevance to other LMICs. This is particularly important since in India, despite increased provision of care, maternal mortality remains high and difficult to address.

### Strengths and limitations

The use of mixed methods to capture the situation regarding obstetricians who did not have regular observable shift handovers is a strength of this study. Other strengths were that all potential participants agreed to take part in the study, and a large number and proportion of patient handovers were observed, exceeding the sample size requirement of 97 patient handovers. The sample size for the government tertiary hospital was small in comparison with the other two hospitals, and although the patterns observed seemed to reflect the other two hospitals with clear points for action, conclusions pertaining to this hospital should be treated with caution.

The challenges of data collection included ensuring that a sample of all handovers were observed, and that enough clinical information had been collected to provide adequate clinical context for each patient being handed over. For the latter, clear and well-defined minimum as well as additional standards for handover were required during data collection. Data collectors also needed to be consistent in their technique and have equal training. This helped to ensure that all the data collected from handovers were comparable, as the process of observation can be complex due to poorly managed handovers and the number of observations. Furthermore, a challenge arose in deciding how to randomly observe and collect data from one handover when there were other handovers occurring in the same speciality simultaneously. Finally, confidentiality needed to be ensured for each institution and staff member being observed. This enabled consent to be obtained adequately.

As with any observational study, a limitation is possible reactivity biases the nurses may have changed their usual behaviour whilst being observed for completion of the data schedule. The direction of the bias is hard to establish as the nurses may have either included more information than they would usually, or made more mistakes than they would ordinarily due to the pressure of being observed. If they included more information than usual due to study observations, our results are of even greater concern since they show that normally very little information would be handed over.

A further limitation is the time that has passed between data collection and publication of results. This should be taken into account when considering generalisability of the findings, as practice may have changed in the intervening time period.

In terms of generalisability, it can probably be assumed that these findings are a best-case scenario for obstetric handovers throughout India as Kerala has the lowest MMR in India [2]. Therefore, it is likely that there is potential for even more improvement in the proportion of women and quantity of information handed over in obstetric care across other less developed states in India. A strength for generalisability is that we had data from 3 types of facilities including a highly regarded private tertiary hospital. However, in India much variation exists between government hospitals and even more so in private hospitals across Kerala, which may lack the degree of regulation and standardization of government hospitals. This may mean that not all private hospitals give such comprehensive handovers, nor will they have a better quality of care compared to tertiary government hospitals [50]. Any interventions should be aimed at both government and private hospitals.

Future research should also be directed at collecting more clinical information on each woman in order for the researchers to stratify whether a patient is high risk and therefore consider if higher-risk patients were given their due attention. This is in addition to minimal standard of handover that all patients and pregnant women should be experiencing during admission. Furthermore it would be important to correlate handover with clinical outcomes to establish the impact of a quality handover on patient outcomes making the results more clinically significant. As noted in the discussion, patient satisfaction is also an important marker of adequate handover, so this should be considered as a primary outcome.

Lastly future research should survey larger and more diverse maternity centres. The focus of this research can be on a more structured assessment of facilitators and barriers to improved quality of care. Future studies can also consider the influence on the quality of handover on both clinical maternal and neonatal outcomes.

## Conclusion

This study in 3 diverse types of hospitals in Kerala, India, showed that the majority of obstetric in-patient women were not being handed over in the secondary hospital between nursing shifts while those who were handed over had inadequate information given in all types of hospital services studied. Nurses carrying out hand over were often distracted or overheard. Despite there being no shift-to-shift handovers for doctors, obstetricians appeared satisfied with information transfer methods available to them; this is worrying given the poor or a lack of handover they experienced. However, most were aware of the importance of information exchange for patient safety and desired more routine, and all wished for information transfer to occur as a team and face-to-face. Although universal awareness of the importance of shift-to-shift handover as well as handover for all women with minimal quality standards (protocol, training and monitoring) seemed to be a high priority in obstetric wards in the settings we studied, it fell far short of what would be considered optimal in terms of patient safety.

## Supporting information

S1 Fig Data schedule. (DOCX)

S2 Fig Interview topic guide. (TIF)

S1 File Qualitative methods. (DOCX)
