# Supplementary material for: Obstetric shift-to-shift handover in Kerala, India: A cross-sectional mixed method study
Source: PLoS One. 2022 May 12;17(5):e0268239. doi: 10.1371/journal.pone.0268239 (PMC9098034; doi:10.1371/journal.pone.0268239)
Supplement: S1 Fig — (DOCX) [file pone.0268239.s001.docx]

**S1 Fig. Data schedule**

**OBSERVATION OF HANDOVER: HANDOVER CHARACTERISTICS (PART ONE, per handover)**

Date __/__/____ Day of week:

Time: Duration (minutes):

Location:

Face-to-face handover : YES/NO If no, method of handover:

No. of patients handed over: No. of patients on ward:

Health care professionals present (job title):

**Please Circle:* Additional Comments:**

Handover not delayed (except in emergencies) YES/NO/NA

Primary person/team responsible for patient identified YES/NO/NA

Junior doctor/nurse participation YES/NO/NA

Information repeated back to ensure accuracy YES/NO/NA

Opportunity for receiving team to ask questions YES/NO/NA

Only standardised medical language was used YES/NO/NA

Hard copy of information alongside verbal handover YES/NO/NA

Hard copy typed, not handwritten YES/NO/NA

Handover free from distractions YES/NO/NA

Handover was not overheard by those not involved YES/NO/NA

**TOTAL /10**

**OBSERVATION OF HANDOVER: PATIENT INFORMATION ITEMS (PART TWO, per patient)**

**Patient had/is having a normal labour YES/NO**

**Patient is stable YES/NO**

**Please Circle:** Additional Comments

**SITUATION /8**

Patient name YES/NO/NA

Patient age YES/NO/NA

Patient location YES/NO/NA

Vital signs YES/NO/NA

Resuscitation status/advanced directive YES/NO/NA

Any specific concerns YES/NO/NA

Key patient values and preferences YES/NO/NA

Special patient needs (e.g. language) YES/NO/NA

**BACKGROUND /10**

Date of admission YES/NO/NA

Brief history YES/NO/NA

Results of physical examinations YES/NO/NA

Diagnosis/active problem list YES/NO/NA

Current medications

Allergies YES/NO/NA

Laboratory results YES/NO/NA

Pending test results YES/NO/NA

Progress during admission YES/NO/NA

Other information from patient charts YES/NO/NA

**ASSESSMENT /3**

Critical assessment of situation YES/NO/NA

Clinical impression YES/NO/NA

Detailed expression of concerns YES/NO/NA

**RECOMMENDATIONS /4**

Management plan YES/NO/NA

Anticipated therapy YES/NO/NA

Suggestions/specifics about timeframe YES/NO/NA

Suggestions/specifics about requests YES/NO/NA

**TOTAL: /25**

**(Version 1.1, 15/01/2015)**

*Note NA in this context means not applicable
