# Supplementary figures and images for: Obstetric shift-to-shift handover in Kerala, India: A cross-sectional mixed method study
Source: PLoS One. 2022 May 12;17(5):e0268239. doi: 10.1371/journal.pone.0268239 (PMC9098034; doi:10.1371/journal.pone.0268239)

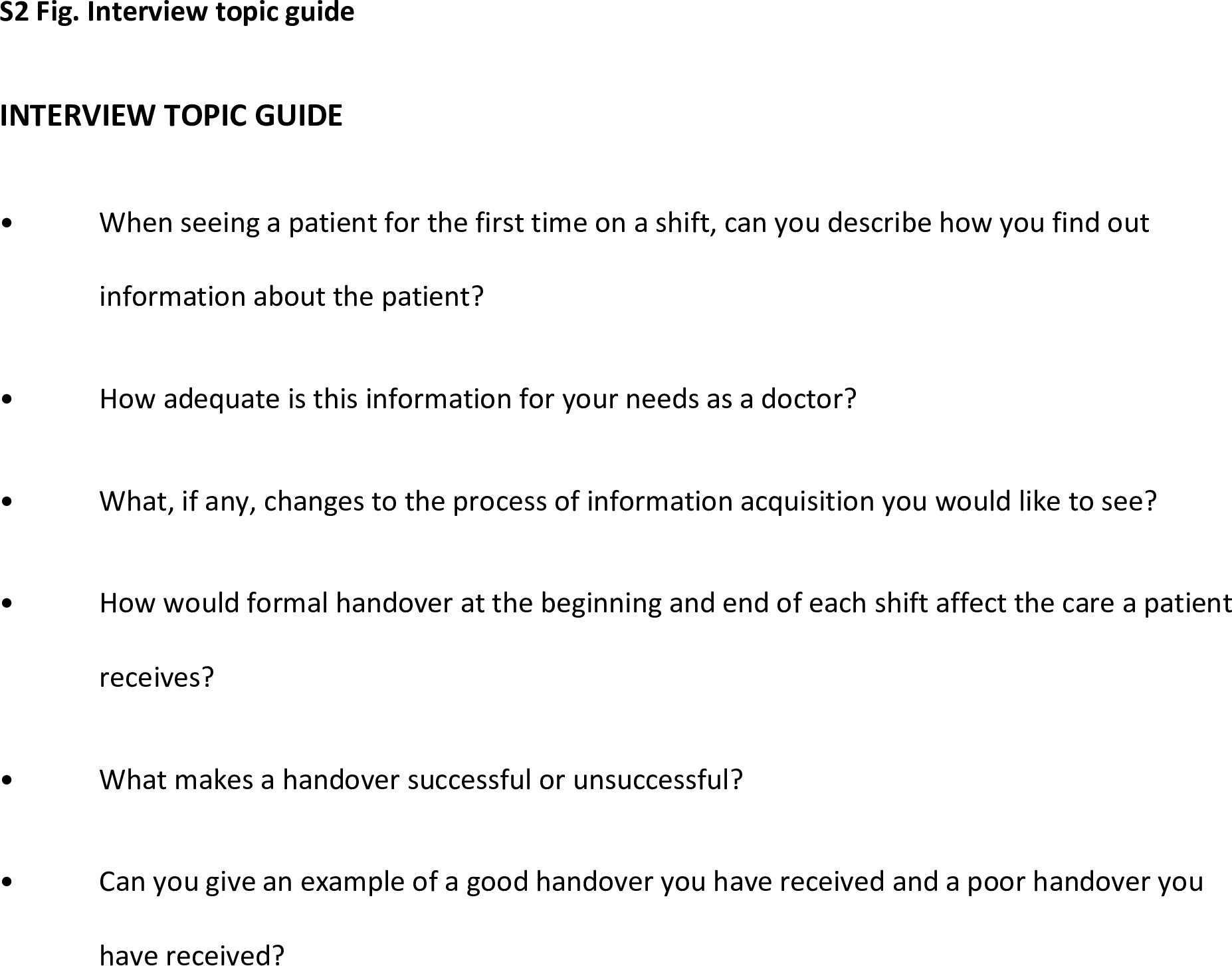

Supplement: S2 Fig — (TIF) [file pone.0268239.s002.tif]
