# Supplementary material for: Obstetric shift-to-shift handover in Kerala, India: A cross-sectional mixed method study
Source: PLoS One. 2022 May 12;17(5):e0268239. doi: 10.1371/journal.pone.0268239 (PMC9098034; doi:10.1371/journal.pone.0268239)
Supplement: S1 File — (DOCX) [file pone.0268239.s003.docx]

**S1 File. Qualitative Methods**

Convenience sampling was used due to the small number of obstetricians who worked in the hospitals. The inclusion criteria was being an obstetrician at one of the study hospitals. The exclusion criteria was being unable to speak fluent English.

The researcher approached the potential participants at a convenient time. They were given participant information sheets and given at least 24 hours to decide if they would like to take part in the research. The participants gave informed, signed consent to be interviewed; none withdrew consent. Interviews were conducted in private locations within the hospitals, and were conducted in English. A translator was present in case the researcher was not understood by the participants.

Open interviews allowed the participants to talk freely about this topic, thus enabling unexpected concepts to emerge and giving the researcher flexibility to probe any unpredicted topics[1,2]. An initial question was always asked: “When seeing a woman for the first time on a shift, can you describe how you find out information about the woman?” A topic guide of opening questions was then used. Participants’ responses were probed and clarified by the researcher. The interviews were transcribed verbatim by the researcher, as soon as possible after the interview. This began the process of familiarisation with the data[3].

A thematic content analysis was performed, in order to describe the situation rather than generate theory. The analysis was conventional (bottom-up) as it was directed by the data. This is because there were no pre-analytic theories on which to base the analysis[4,5]. Analysis commenced once all interviews had been conducted. The data was analysed using Braun and Clarke’s six step process[5].Firstly, transcripts were read for familiarisation and idea generation. Data was then coded systematically. All data was coded regardless of its apparent relevance. More than one code was given, if appropriate[4]. In the third step, unrefined themes were generated by gathering together codes with similar meanings. The themes were then reviewed using a mind map to ensure the meaning of the whole data set was reflected and the situation was fully described as told by the participants. The fifth stage was to name and define themes. The final stage of analysis involved selecting quotes to exemplify the themes.

To reduce the risk of bias, member validation was performed via email. The participants were asked to comment upon whether the conclusions drawn represent a reasonable summary of their experiences. Deviant case analysis was performed in which data to disprove the conclusions reached was actively sought.

**References**

1. Britten N. Qualitative interviews in medical. Br Med J [Internet]. 1995;311(6999):251–3. Available from: https://www.jstor.org/stable/29728175

2. Ives J. Qualitative Methodology. In: Walker D, editor. An Introduction to Health Services Research: A Practical Guide. Sage; 2014. p. 99–114.

3. Ziebland S, McPherson A. Making sense of qualitative data analysis: An introduction with illustrations from DIPEx (personal experiences of health and illness). Med Educ [Internet]. 2006;40(5):405–14. Available from: https://scite.ai/reports/10.1111/j.1365-2929.2006.02467.x

4. Bowling A. Research methods in health : investigating health and health services [Internet]. 2nd ed. Buckingham: Open University Press; 2002. Available from: https://edisciplinas.usp.br/pluginfile.php/4374657/mod_resource/content/1/Research Methods in Health.pdf

5. Braun V, Clarke V. Using thematic analysis in psychology. Qual Res Psychol [Internet]. 2006;3(2):77–101. Available from: https://www.tandfonline.com/doi/abs/10.1191/1478088706qp063oa
